# Application of Physical Examination Data on Health Analysis and Intelligent Diagnosis

**DOI:** 10.1155/2021/8828677

**Published:** 2021-06-13

**Authors:** Xiao-Ling Wang, Jun Liu, Zi-Qi Li, Zhi-Lin Luan

**Affiliations:** ^1^Department of Physical Education, School of Civil Engineering, Dalian University of Technology, Dalian 116024, China; ^2^Advanced Institute for Medical Sciences, Dalian Medical University, Dalian 116044, China

## Abstract

Analysis and diagnosis according to the collected physical data are an important part in the physical examination. Through the data analysis of the physical examination results and expert diagnoses, the physical condition of a specific physical examination unit can be achieved which may guide individual health development. However, in general, the application of physical examination data is insufficient in most of the current physical examination organizations. Therefore, in the present study, statistical analysis and intelligent diagnosis were applied to maximize the utilization of physical examination data. The physical examination data collected from different departments of Dalian University of Technology were statistically analyzed and then synthesized for stimulating the thinking mode and knowledge framework of medical experts by a learning model on machine, resulting in the construction of an intelligent physical examination diagnosis method with 93.4% accuracy confirmed by experts. In conclusion, a potential artificial intelligence model of psychical examination data on health analysis and intelligent diagnosis was established, which may become more and more accurate with data accumulation in the near future.

## 1. Introduction

Health is an inevitable requirement for promoting the all-round development of human beings and a basic condition for economic and social development. The “Healthy China 2030 Planning Outline” clarifies the requirements to promote the integration of sports and medicine to accelerate the development of disease management system and health service mode, which may play an active role in health promotion and chronic disease prevention and rehabilitation. In China, with rapid social development, public health literacy and disease prevention awareness improve persistently and rapidly, and regular physical examination has become an important part in public health care. Therefore, a large amount of physical examination data has been generated, including physical examination raw data and expert advice [1–4]. At present, most physical examination organizations have not sufficiently used the large amount of physical data, leading to data waste and the efficiency of physical examination. Valid and sufficient application of physical examination data combined with artificial intelligence methods can make more convenient and accurate judgment on the physical condition of each examinee [5–7].

As an intelligent system with a large amount of experience and expertise accumulated in a specific field, expert system based on artificial intelligence refers to the knowledge system established by the expert's existing knowledge system [8–14]. The system uses the technology and theory in the field of artificial intelligence to solve problems in similar fields by simulating the problem-solving thinking mode of experts. Under some circumstances, the problem-solving ability of the expert system is equal to or even beyond that of human experts. The expert system has already become a program system for collecting and accumulating professional knowledge and experience in some fields. It applies many artificial intelligence technologies to simulate the thinking mode of human experts when solving problems and even solves various problems difficult for experts [15–17].

In some previous studies, expert systems have been applied in many fields, such as justice, medical treatment, and art. Tan [18] briefly introduced the development and basic structure of the expert system. Then, from the perspective to enable technology, current expert systems have been classified into four elaborate expert systems: the Rule-Based Expert System, the Framework-Based Expert System, the Fuzzy Logic-Based Expert System, and the Expert System Based on Neural Network. Magrabi et al. [19] discussed the prospects of the application of artificial intelligence technology in clinical decision-making in 2019, which indicated that after establishing an effective expert evaluation framework, artificial intelligence can assist medical institutions in clinical decision-making.

In the present study, to achieve more effective and comprehensive utilization of physical examination data and improve the uneconomic diagnosis by manual labor, we conducted a statistical analysis of physical examination data in a population and then applied machine learning methods to integrate the existing physical examination data and diagnosis results to simulate the diagnosis logic of medical experts for potential intelligent health diagnosis.

## 2. Materials and Methods

### 2.1. Processing of the Physical Examination Raw Data

The raw data used in the present study was from the Health Center of Dalian University of Technology. Seven physical examination indicators were included and checked for each examinee (Table 1). Based on the results of the physical examination, the data of 1,352 examinees from ten departments of Dalian University of Technology (Table 2) was finally included for statistical analysis. The data for a specific indicator was considered as reaching the standard according to the National Physical Examination Standards of China. The statistics were the percentages of the individuals reaching the standard values for these indicators, and Spearman's rank correlation coefficient was applied to compare age, department, and physical examination indicators.

### 2.2. Intelligent Diagnosis

There were three steps for our intelligent diagnosis. In the first step, we used a feature encoding technique to convert physical examination indicators into feature codes for training and expert opinions into corresponding labels. The second step was training the XGBoost machine learning model. The third step was intelligently diagnosing the examinee by the trained model.

#### 2.2.1. Feature Encoding Technology

We used a feature encoding technology to convert physical examination indicators into numerical indicators, and numbers represented the levels of the different physical examination indicators. The raw data was shown in text form (Table 3). The textual physical examination results were converted to numerical values by scoring the results from 1 to 4 or 5 depending on how many levels of the scored parameter are there.  Table 4 showed the results after encoding.

Medical experts were invited to diagnose the samples, and the common medical diagnosis opinions were displayed one by one in a certain order. The experts used 0 and 1 as the examination results for recommendation or prohibition, respectively, which can be counted in order to obtain the binary code. Then, the binary code was converted to decimal code as the category label of this sample. At last, the physical examination scores and category labels were used as data for training.

#### 2.2.2. XGBoost Model

Extreme Gradient boosting (XGBoost) [20] is a machine learning system based on gradient boost [21] with the feature of insensitivity to missing values which is suitable for our data processing due to the large number of missing values in the physical examination data. The tree ensemble model used by XGBoost is a set of classification and regression trees (CART) [22]. This enhancement method that uses trees as a basic learner is called tree enhancement. Because one tree may not be enough to obtain good results, multiple CART are usually used at the same time. The final prediction is the sum of the scores of each CART. The model can be written as

(1) $$\begin{array}{c} \hat{yi}=\varphi \left(xi\right)=\overset{K}{\underset{k=1}{\sum }}f_{k}\left(X_{i}\right),\;f_{k}\in F.\text{s} \end{array}$$

In this equation (1), *F* = {*f*(*x*) = *w*_*q*(*x*)_}(*q* : ℝ^*m*^⟶*T*, *w* ∈ ℝ^*T*^) is the space of the return tree, *q* represents a mapping relationship, which can map samples to corresponding leaf nodes, *T* represents the number of leaves in the tree, *w* represents the weight of the leaves, and *K* represents the number of trees.

Equation (2) is the optimized objective function, and the parameter *f*_*t*_ is added to help minimize the objective function, where ${{\overset{\wedge }{y}}_{i}}^{\left(t-1\right)}$ means the predicted value of test sample *i* at *t* − 1 iteration, $l\left(y_{i},{{\overset{\wedge }{y}}_{i}}^{\left(t-1\right)}\right)$ means loss function, and *Ω* is a regularization term.

(2) $$\begin{array}{c} L^{\left(t\right)}=\overset{n}{\underset{i}{\sum }}l\left(y_{i},{{\overset{\wedge }{y}}_{i}}^{\left(t-1\right)}+f_{t}\left(x_{i}\right)\right)+\Omega \left(f_{t}\right). \end{array}$$

The regularization term is calculated using equation (3) to control the fitting variance and the flexibility of the learning task, so as to obtain a stronger generalization ability and also avoid overfitting of the model by controlling the complexity of the model.

(3) $$\begin{array}{c} \Omega \left(f\right)=y^{T}+\frac{1}{2}\lambda {\left\|w\right\|}^{2}. \end{array}$$

#### 2.2.3. Diagnosis and Prediction

**Figure 1:**
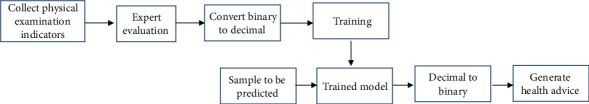
Algorithm flow.

We encoded the new physical examination indicators and input them into the trained XGBoost model. After the labels were output, the output labels were binary-decoded to obtain the diagnosis opinion of the medical experts. The algorithm flow is shown in Figure 1.

## 3. Results

### 3.1. Statistics of Physical Examination Indicators

**Figure 2:**
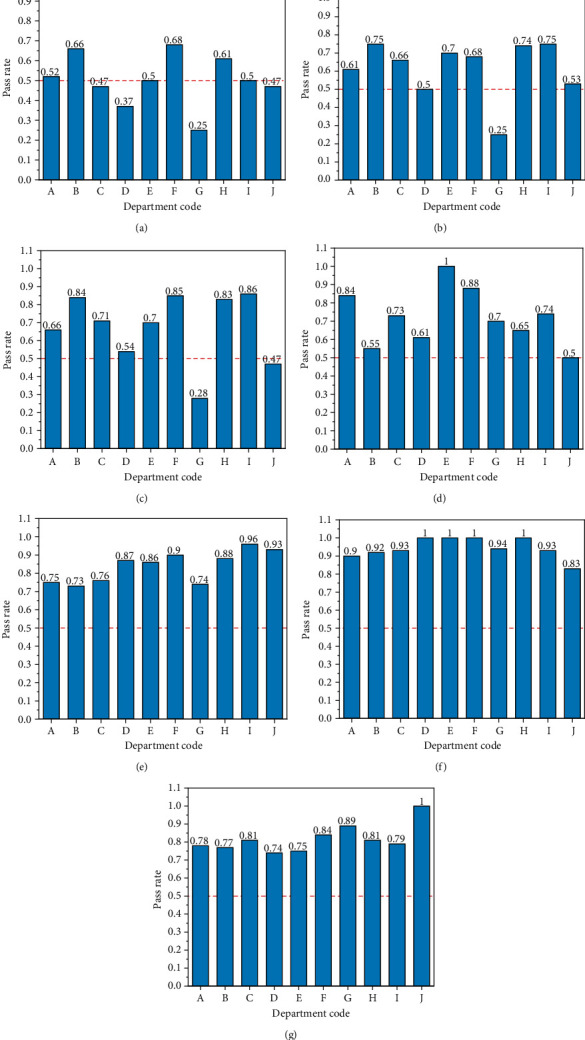
Percentages of the individuals reaching the standard values for the seven physical examination indicators: (a) BMI; (b) body fat percentage; (c) visceral fat grade; (d) bone density *T* value; (e) heart function; (f) blood vessel elasticity; (g) vascular obstruction.

**Figure 3:**
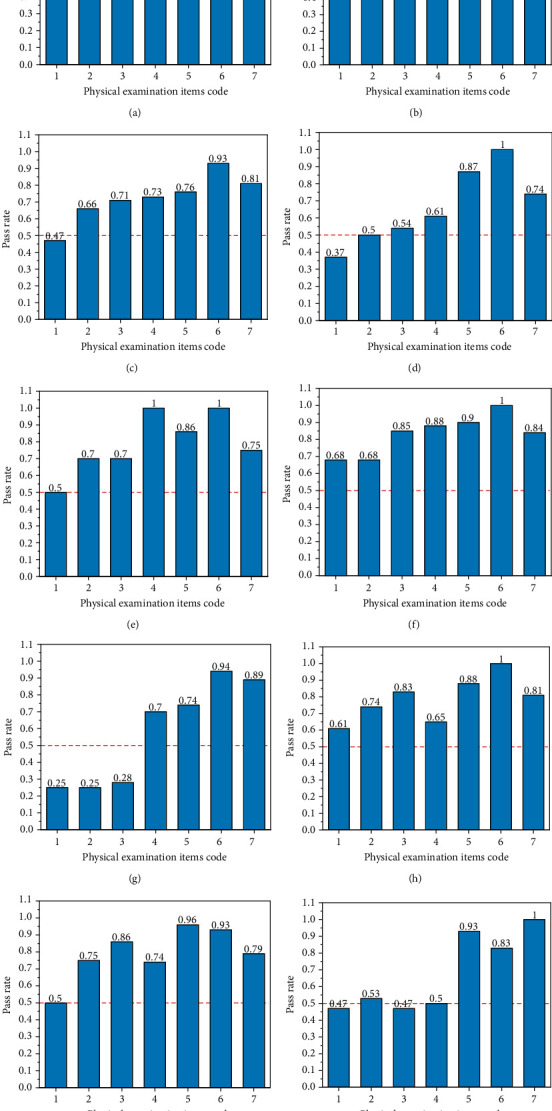
Percentages of the individuals reaching the standard values for the seven physical examination indicators in different departments: (a) administrative staff; (b) library; (c) School of Distance and Continuing Education; (d) Faculty of Vehicle Engineering and Mechanics; (e) School of Mechanical Engineering; (f) Physical Education Department; (g) 2018 Xinjiang Class; (h) School of Chemical Engineering; (i) High School of Dult; (j) School of Physics.

**Figure 4:**
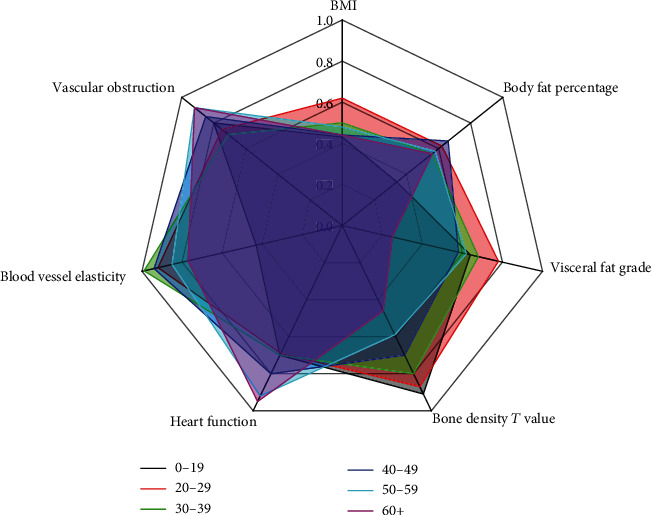
Analysis of the indicators in different age groups.

The percentages of the individuals reaching the standard values for the seven indicators are summarized in Figure 2. The statistical variance and average of the seven indicators are shown in Table 5. The statistical results of the seven indicators in different departments are shown in Figure 3. The statistical variance and average of the percentages in different departments are shown in Table 6. The statistical analysis of the indicators in different age groups is shown as a radar chart in Figure 4. The Spearman rank correlation coefficients of age, department, and examination indicators are shown in Table 7.

### 3.2. Intelligent Diagnosis

**Figure 5:**
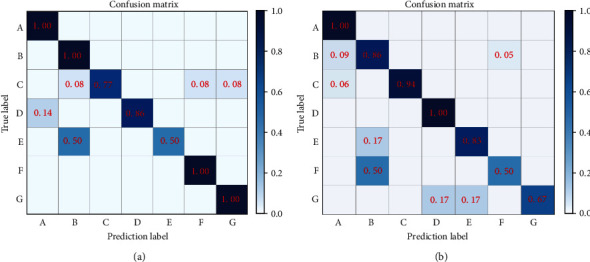
Training results: (a) simulated training results of expert A; (b) simulated training results of expert B.

**Figure 6:**
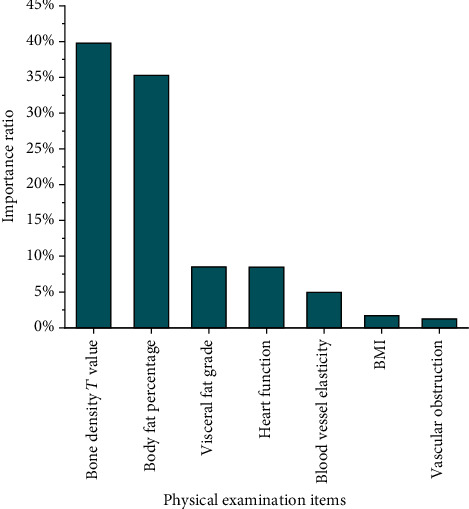
Importance ranking of expert's training results.

After 100 training steps, the training accuracy of the model was 93.4%. The normalized confusion matrix obtained is shown in Figure 5, where A~G represented the diagnosis opinions under seven different conditions. The diagonal line presented a high-density area, and a small number of diagnostic opinions showed low accuracy rates, but all above 50%. The reason for the low accuracy is the relatively low number of samples, which led to the inability of the model to accurately simulate the expert's diagnostic logic. According to the results of the importance ranking in Figure 6, two physical examination indicators, body fat rate and bone density *T* value, showed relatively large weights, while the weights of other indicators were relatively small, indicating that experts mainly referred to these two indicators, the levels of which have a greater impact on body health.

## 4. Discussion

In the present study, we statistically analyzed seven indicators of physical examination. By comparison, we found that four indicators, body mass index (BMI), body fat percentage, visceral fat grade, and bone density, showed relatively poor results with low variance and average standard rate, especially in Xinjiang Class. These four indicators are associated with daily exercise habits, which suggested that the exercise supervision and guidance were lacking and required more attention. On the other hand, the variances of cardiac function, vascular elasticity, and vascular obstruction were relatively small, and the standard rate was relatively good. These three parameters are generally related to diet, indicating that the university paid enough attention to diet, and the risk of cardiovascular and cerebrovascular diseases in the collected population was relatively low.

The statistical results showed that the Physical Education Department had the highest standard rate, 0.83, and the distribution of the indicators in this department was relatively stable, which may have resulted from more regular and intensive physical exercise. The average standard rate for the School of Chemical Engineering, the School of Science and Technology, the School of Distance and Continuing Education, the Dalian Institute of Technology, the School of Mechanical Engineering, and the library all exceeded 0.7, indicating that the physical examination of most the employees in these departments was qualified. The indicator variances of the School of Mechanical Engineering were relatively large, reaching 0.17. The standard rates for obesity-related physical examination indicators were relatively low, and the standard rates for indicators related to heart and blood vessel function were high, indicating that the examinees in the School of Mechanical Engineering lack physical exercise. The College of Transportation, the College of Physics, and the 2018 Xinjiang Class had relatively low standard rates, 0.66, 0.68, and 0.58, respectively, and relatively large distribution range for most parameters. The possible reason for these results was the extremely low standard rates of the BMI, body fat percentage, and visceral fat level in these three departments, indicating that the subjects in these three departments need to strengthen physical exercise and carry out further testing and treatment for those who do not meet the standards.

The radar chart showed that the employees over the age of 60 in Dalian University of Technology had poor performance in bone density with a standard rate of ~0.43. The bone density generally decreases along with age which requires medication for improvement in elder people. However, as the work pressure is relatively low for the elderly employees in the university, they showed relatively better performance of the heart function and blood vessel obstruction with a standard rate over 0.8. On the other hand, the performance of visceral fat level and BMI was relatively poor for these elderly employees with a standard rate around 0.4, which may be associated with lacking of exercise. These indicators showed relatively even distribution in middle-aged employees with moderate standard rates. The body fat rate among the employees between 0 and 19 years old showed relatively low value compared to the other groups of employees with a standard rate around 0.36, indicating that obesity among young people is more obvious. In terms of individual indicators, with the age, the value of bone density declines in a gradient way. Therefore, bone density is a valid indicator for age. In terms of vascular elasticity, the gap between different age groups was small with no obvious alteration with age.

Figure 5 shows that the prediction effect of the model is good. In the 7 types of treatment plans, the average prediction accuracy rates is 93.4%. With the increase of samples, the accuracy of the model will be further improved. In a few treatment options, the prediction accuracy rate is lower. The reason is that BMI, body fat percentage, and visceral fat grade in the original data were relatively highly correlated. They all indicate fat and thin indicators, so when scoring, the indicators that experts focus on will be slightly different, leading to a decrease in sample regularity and attenuation of the model's predictive ability.

According to the results of the importance ranking shown in Figure 6, body fat rate and bone density *T* value were given relatively large weights, while the weights of other physical examination indicators were relatively small, indicating that the body fat percentage and bone density *T* value of the examinees in the sample set distributed more balance.

## 5. Conclusions

In the present study, a statistical analysis of the collected physical examination data was performed which showed the distributions and differences of seven physical examination indicators in different departments of Dalian University of Technology. According to the results, an artificial intelligence-based health diagnosis method was proposed. This method organized the diagnosis opinions of the medical experts into a data set and used the XGBoost classification model to train the data set which simulated the expert's diagnosis logic for intelligent diagnosis with an accuracy rate of 93.4%. This new model may become more and more accurate with data accumulation in the near future.

## Figures and Tables

**Table 1:** Codes for the physical examination indicators checked in the study.

Code	Physical examination indicators
1	Body mass index (BMI)
2	Body fat percentage
3	Visceral fat grade
4	Bone density *T* value
5	Heart function
6	Blood vessel elasticity
7	Vascular obstruction

**Table 2:** Information of the departments involved in the study.

Code	Department	Number of people
A	Administrative staff	272
B	Library	209
C	School of Distance and Continuing Education	187
D	Faculty of Vehicle Engineering and Mechanics	151
E	School of Mechanical Engineering	137
F	Physical Education Department	121
G	2018 Xinjiang Class	88
H	School of Chemical Engineering	65
I	High School of Dult	64
J	School of Physics	58

**Table 3:** Physical examination raw data.

1	2	3	4	5	6	7
Fat	Fat					
Appropriate	Standard health	Normal				
Fat	High fat	High	Soft	Critical state	Bone normal	Good
Appropriate	Standard health	Normal	Standard	Normal	Bone normal	Medium
…	…	…	…	…	…	…
Appropriate	Too low	Normal				

**Table 4:** Coding result.

1	2	3	4	5	6	7
4	4					
3	3	3				
4	5	5	2	4	3	1
3	3	4	4	3	3	3
…	…	…	…	…	…	…
3	1	4				

**Table 5:** Statistics of the seven physical examination indicators.

Physical examination index	BMI	Body fat percentage	Visceral fat grade	Bone density T value	Heart function	Blood vessel elasticity	Vascular obstruction
Average value	0.50	0.62	0.67	0.72	0.84	0.95	0.82
Standard deviation	0.12	0.15	0.18	0.15	0.08	0.05	0.07

**Table 6:** Statistics on the percentages of the individuals reaching the standard values in different departments.

Department name	Average value	Standard deviation
Administrative staff	0.72	0.12
Library	0.75	0.11
School of Distance and Continuing Education	0.72	0.13
Faculty of Vehicle Engineering and Mechanics	0.66	0.2
School of Mechanical Engineering	0.79	0.17
Physical Education Department	0.83	0.11
2018 Xinjiang Class	0.58	0.29
School of Chemical Engineering	0.79	0.12
High School of Dult	0.79	0.14
School of Physics	0.68	0.22

**Table 7:** Spearman's rank correlation coefficient.

	Age	Departments	1	2	3	4	5	6	7
Age	1	0.245	0.165	0.245	0.173	-0.244	0.207	-0.174	0.121
Departments	0.245	1	-0.012	-0.029	-0.06	-0.152	0.033	0.023	0.18
1	0.165	-0.012	1	0.804	0.699	-0.181	-0.172	-0.131	0.01
2	0.245	-0.029	0.804	1	0.689	-0.166	-0.042	-0.097	0.123
3	0.173	-0.06	0.699	0.689	1	-0.139	-0.087	-0.077	-0.028
4	-0.244	-0.152	-0.181	-0.166	-0.139	1	-0.13	0.191	-0.017
5	0.207	0.033	-0.172	-0.042	-0.087	-0.13	1	-0.013	0.185
6	-0.174	0.023	-0.131	-0.097	-0.077	0.191	-0.013	1	0.018
7	0.121	0.18	0.01	0.123	-0.028	-0.017	0.185	0.018	1

## Data Availability

All data generated or analyzed during this study are included in this published article.
